# Structure, (governance) and health: authors reply

**DOI:** 10.1186/1472-698X-7-1

**Published:** 2007-01-26

**Authors:** Anatole S Menon-Johansson

**Affiliations:** 1 St. Stephen's Centre, Chelsea & Westminster Hospital, 369 Fulham Road, London, SW10 9NH, UK

## Abstract

This is a reply to the paper entitled Structure, (governance) and health: an unsolicited response by Daniel D Reidpath and Pascale Allotey

## Text

I would like to thank Drs Reidpath and Allotey for their analysis [1] of some of the variables described in the tables of the initial paper [2] and their use of other health and economic data to demonstrate correlations with governance.

HIV infection was chosen as the focus in the original work because in some sub-Saharan African countries this single disease has become ubiquitous. This is also the case within high risk groups across the world from men who have sex with men in London to sex workers in Mumbai. Unlike geographically determined disease such as Chagas disease and Guinea worm infections cited by Reidpath and Allotey, HIV is found in every nation and the epidemic in many populous countries is becoming generalised (> 1% prevalence).

**Figure 1:**
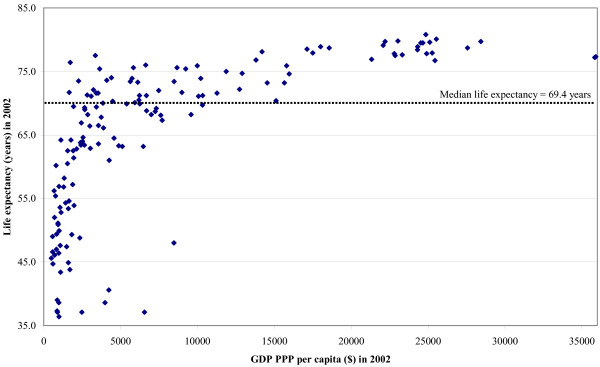
A scatter graph of GDP-PPP and life expectancy for 149 countries in 2002.

Reidpath and Allotey demonstrate a strong correlation between healthy life expectancy and gross domestic product per capita corrected for purchaser power parity (GDP-PPP) with governance [1]. They further show that governance and GDP-PPP variables are themselves interrelated. Tables 5–7 in the initial paper, showing health and economic data for 149 countries grouped by low, middle and high governance ranking, highlighted some of the structural challenges these countries face. The small GDP-PPP in low governance countries is compounded by poorer distribution of wealth. The impact of GDP-PPP on life expectancy in 2002 is demonstrated in Figure 1, using data from the additional file included with the original paper [2]. In poor countries, small increases in wealth produce a dramatic increase in life expectancy with diminishing returns above the median life expectancy of 69.4 years in 2002.

In addition to wealth, these low governance countries have fewer physicians per population and less investment in health and education in relation to military spending. The residents of poorer countries are more reliant on their governments to support public investment in healthcare since they are less able to purchase care in the private sector. The maternal mortality ratio was included in these tables to illustrate the devastating impact of these interrelated variables.

Despite the quality concerns of the aggregate data available, sufficient HIV variance was explained by governance to reject the null hypothesis using non-parametric statistical analysis. The unprecedented international response to the HIV pandemic is highly dependent upon the co-operation of local and national government thus warranting an analysis of the relationship between HIV and governance. Again it is important to stress that 'horizontal' capacity building strategies are vital if HIV/AIDS is going to be effectively managed in nations with limited healthcare infrastructure and underlying poor health.

Only with time and additional governance and health data will it be possible to determine if they are causally related. If they are, then it is likely that some dimensions of governance are going to be more influential than others on healthcare delivery. Consequently it is important that each dimension of governance continues to be analysed separately. Ideally future analysis should be controlled for by wealth (GDP-PPP) and its distribution (GINI index) in order to understand the role of governance more clearly.

I would like to thank the authors again for their unsolicited response and encourage future researchers to focus on governance and its impact on health care policy and delivery not only in regards to chronic treatable disease, such as HIV, but also for more finite challenges such as maternal health.

## Pre-publication history

The pre-publication history for this paper can be accessed here:


